# My Hypertension Education and Reaching Target (MyHEART): Development and Dissemination of a Patient-Centered Website for Young Adults with Hypertension

**DOI:** 10.2196/cardio.8025

**Published:** 2017-09-25

**Authors:** Heather M Johnson, Jamie N LaMantia, Colleen M Brown, Ryan C Warner, Laura M Zeller, Ryan C Haggart, Keven Stonewall, Diane R Lauver

**Affiliations:** ^1^ Department of Medicine School of Medicine and Public Health University of Wisconsin-Madison Madison, WI United States; ^2^ Health Innovation Program School of Medicine and Public Health University of Wisconsin-Madison Madison, WI United States; ^3^ Department of Population Health Sciences School of Medicine and Public Health University of Wisconsin-Madison Madison, WI United States; ^4^ Department of Counselor Education and Counseling Psychology Marquette University Milwaukee, WI United States; ^5^ Department of Preventive Cardiology University of Wisconsin Hospitals and Clinics Madison, WI United States; ^6^ School of Nursing University of Wisconsin Madison, WI United States

**Keywords:** hypertension, young adults, World Wide Web, quality improvement, patient engagement

## Abstract

**Background:**

Young adults (18 to 39 years old) with hypertension have the lowest rates of blood pressure control (defined as blood pressure less than 140/90 mmHg) compared to other adult age groups. Approximately 1 in 15 young adults have high blood pressure, increasing their risk of future heart attack, stroke, congestive heart failure, and/or chronic kidney disease. Many young adults reported having few resources to address their needs for health education on managing cardiovascular risk.

**Objective:**

The goal of our study was to develop and disseminate a website with evidence-based, clinical information and health behavior resources tailored to young adults with hypertension.

**Methods:**

In collaboration with young adults, health systems, and community stakeholders, the My Hypertension Education and Reaching Target (MyHEART) website was created. A toolkit was also developed for clinicians and healthcare systems to disseminate the website within their organizations. The dissemination plan was guided by the Dissemination Planning Tool of the Agency for Healthcare Research and Quality (AHRQ).

**Results:**

Google Analytics data were acquired for January 1, 2017 to June 29, 2017. The MyHEART website received 1090 visits with 2130 page views; 18.99% (207/1090) were returning visitors. The majority (55.96%, 610/1090) approached the website through organic searches, 34.95% (381/1090) accessed the MyHEART website directly, and 5.96% (65/1090) approached through referrals from other sites. There was a spike in site visits around times of increased efforts to disseminate the website.

**Conclusions:**

The successfully implemented MyHEART website and toolkit reflect collaborative input from community and healthcare stakeholders to provide evidence-based, portable hypertension education to a hard-to-reach population. The MyHEART website and toolkit can support healthcare providers’ education and counseling with young adults and organizations’ hypertension population health goals.

## Introduction

### Prevalence of Hypertension among Young Adults

Uncontrolled hypertension among young adults (18 to 39 year-olds) [1] is an enormous public health burden [2,3]. In the United States, over 10 million 18 to 39 year-olds (1 in 5 men; 1 in 6 women) have hypertension [4-7], increasing their risk of premature heart failure, stroke, and chronic kidney disease [4,8-11]. Young adults with hypertension have a high lifetime risk for cardiovascular disease due to the longer exposure to high blood pressures and ongoing risk of organ damage [11-17]. Hypertension control reduces morbidity, mortality, and future healthcare costs [18-22]. Yet, only 40% of young adults with hypertension in the United States have achieved blood pressure control (defined as a blood pressure less than 140/90 mmHg) [23-27]. Our prior research demonstrated that within 1 year of developing hypertension, almost half of young adults do not receive guideline recommended lifestyle counseling [28]. Young adults are also less likely to receive a hypertension diagnosis and, if necessary, medication initiation compared to middle-aged and older adults [29,30].

### Hypertension Control Barriers among Young Adults

To further understand barriers to young adults achieving hypertension control, we engaged racially and ethnically diverse young adults in 6 focus groups and conducted one-on-one interviews with primary care providers [31,32]. Two focus groups were conducted at each site: 1 academic, 1 urban, and 1 rural healthcare system [31,32]. The young adult respondents identified hypertension education topics that were not commonly addressed in current educational materials [32]. Young adult respondents shared their preferred social media channels and requested Web-based education to provide flexible access to hypertension information “when they wanted it” [32]. Primary care providers shared similar views of lacking hypertension materials and/or the time for extended education for young adults [31]. Both groups also highlighted other common barriers (eg, transportation, work-life balance, financial limitations) to hypertension care delivery. The combined qualitative and quantitative data highlighted the need to provide a website tailored to young adults with hypertension.

Prior studies demonstrated that when patients with hypertension receive health education targeted to their needs, their self-management of hypertension improves (eg, behavior changes, home blood pressure monitoring) [33]. In addition, patient education should provide a sense of personal medical empowerment to promote, initiate, and maintain health behavior changes [32,34]. Finally, hypertension education can serve as a bridge between clinic visits. To address an unmet need in the delivery of hypertension care for young adults, we developed the My Hypertension Education and Reaching Target (MyHEART) program, a young adult hypertension education program.

### Rationale for Development of the MyHEART Website

The aims of MyHEART are to (1) decrease barriers to young adult hypertension care delivery; and (2) improve hypertension control in this hard to reach population. It is known that website education alone is insufficient for long-term health behavior change; however, it can be effective as an additional component to ongoing hypertension control initiatives [35]. Therefore, the goals of the MyHEART website [36] are to (1) be a portable resource for young adults’ questions and challenges with managing blood pressure; and (2) supplement the hypertension clinical care and education of healthcare teams and organizations. The aims of this proposal were to (1) develop the architectural structure of the MyHEART website through community engagement partnerships; and (2) launch and disseminate the MyHEART website to clinicians, healthcare systems, and community organizations committed to hypertension control.

## Methods

### Ethics

Prior studies [31,32,37] that informed the MyHEART website development were approved by the University of Wisconsin-Madison Health Sciences Institutional Review Board (IRB) and informed consent was obtained from patient and clinician stakeholders. Neither IRB approval nor written consent were needed to design or implement this website because the data that informed MyHEART development was already described in the original IRB submission for the prior studies.

### MyHEART Website Development

#### Community Stakeholders

Our stakeholders consisted of 38 young adult patients with a mean age of 26.7 (SD 9.6) years old and were 34% (13/38) male, 45% (17/38) Black, and 42% (16/38) with 1 or more years of college [32]. In addition, there were 15 primary care clinicians [31] and 3 hypertension quality improvement teams across multiple healthcare systems. The Wisconsin Network for Research Support (WINRS) is a community and patient engagement resource based at the University of Wisconsin-Madison School of Nursing. WINRS developed the Community Advisors on Research Design and Strategies (CARDS), an innovative consultation service that engages lay community members. CARDS includes members from diverse backgrounds, including underrepresented communities and “hard to reach” populations. Members are trained to (1) review project materials (eg, websites, survey questions, mobile phone apps); and (2) provide unique feedback for research, education, and outreach. For our website development, 10 to 12 CARDS members were engaged monthly for 6 months, either in-person or by electronic communication, for feedback on content, architecture, and dissemination plans [38]. In addition, there were 2 90-minute meetings with the CARDS group to discuss updated MyHEART versions and pilot test the user-interface, website usability, and technological options for accessing Web information (eg, mobile phone, tablet).

#### Website Architecture

We built the website in partnership with the University of Wisconsin Health Innovation Program (HIP) using the Drupal 7 Content Management Framework (TurnKey Linux). Based on stakeholder input, the MyHEART website has the following 3 main categories (Multimedia Appendix 1): (1) defining blood pressure and understanding high blood pressure; (2) information on initiating and maintaining behaviors to control blood pressure; and (3) relevant resources, such as exercise options, questions for clinicians, and recipes via website links to the American Heart Association (AHA), Centers for Disease Control (CDC), and National Institutes of Health (NIH). We also feature peer-reviewed publications on cardiovascular health in young adults from established scientific resources (eg, NIH, CDC, AHA) because young adult focus group informants requested to be kept up-to-date [32]. A Twitter feed (@MyHeartMyChoice) was included as another means of sharing important health topics with young adults. A discussion forum is being designed and will be added in the future as the MyHEART program staff expands to support the exchange of ideas.

#### Iterative Website Design Process

The website’s architectural structure was cyclically evaluated by the lay advisory group (CARDS), information technology specialists, and clinical content experts (ie, physicians, nurses) using established categories: internal reliability, external security, content usefulness, navigation usability, and system interface attractiveness [39,40]. A sample of the detailed notes on MyHEART’s architectural structure is in shown in Multimedia Appendix 2; CARDS lay advisory group full meeting notes are available upon written request to the corresponding author. The educational content for the website was formatted with a Flesch-Kincaid readability of the 6th grade or less [41]. Website edits continued over 12 months, with testing on desktop and mobile computer devices, until the final iteration was launched in January 2017.

#### Website Toolkit

A toolkit was developed for the MyHEART website to assist clinicians and healthcare systems with incorporating the website in clinical practice and community outreach. The toolkit provides customizable materials and Web and social media communication drafts to share the website with members of their organization (Multimedia Appendix 3).

### Website Dissemination

The Dissemination Planning Tool of the Agency for Healthcare Research and Quality (AHRQ) [42] was used to outline and navigate the dissemination plan that was started in February 2017. The website and toolkit were first disseminated through HIPxChange [43] in association with the University of Wisconsin HIP. In late 2012, HIP launched HIPxChange to disseminate evidence-based programs, tools, and other materials for free to the public [44]. The goal of HIPxChange is to accelerate the translation of new and existing knowledge into clinical practice to improve healthcare delivery and health outcomes.

Additional dissemination avenues included clinician notifications, academic and healthcare marketing teams, university/campus health centers, research communities, and public community announcement boards (eg, grocery stores, coffee shops, etc). Regional dissemination efforts include the Wisconsin Collaborative for Healthcare Quality (WCHQ). This is a voluntary consortium of 37 Wisconsin healthcare organizations (physician groups, hospitals, health plans) that has led the nation in measuring and improving healthcare quality for multiple chronic conditions [45,46]. Social media streams (eg, Facebook, Twitter, health blogs) have been activated.

## Results

**Figure 1:**
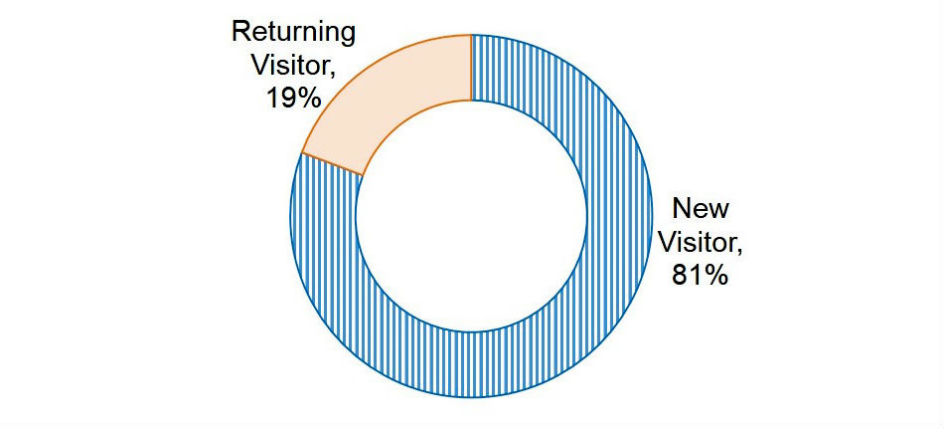
Percentage of new versus returning visitors to the MyHEART website from January 1, 2017 to June 29, 2017 (N=1090).

Google Analytics data were acquired for January 1, 2017 to June 29, 2017. In this time, the MyHEART website received a total of 1090 visits, with an average of 1.95 pages/session (range 1 to 7 pages/session for 95% of users). The number of site visits were in line with our expectations during this implementation period. Among the site visitors, 81.01% (883/1090) were new visitors (Figure 1). The majority (55.96%, 610/1090) approached the website through organic searches, 34.95% (381/1090) accessed the MyHEART website directly, and 5.96% (65/1090) approached through referrals from other sites. Overall, 40 sessions (3.67%, 40/1090) were referred from social media; 23 (2.11%, 23/1090) from Twitter, and 17 (1.56%, 17/1090) from Facebook, with Facebook demonstrating a greater number of unique visitors (10) than Twitter (2 unique visitors).

**Figure 2:**
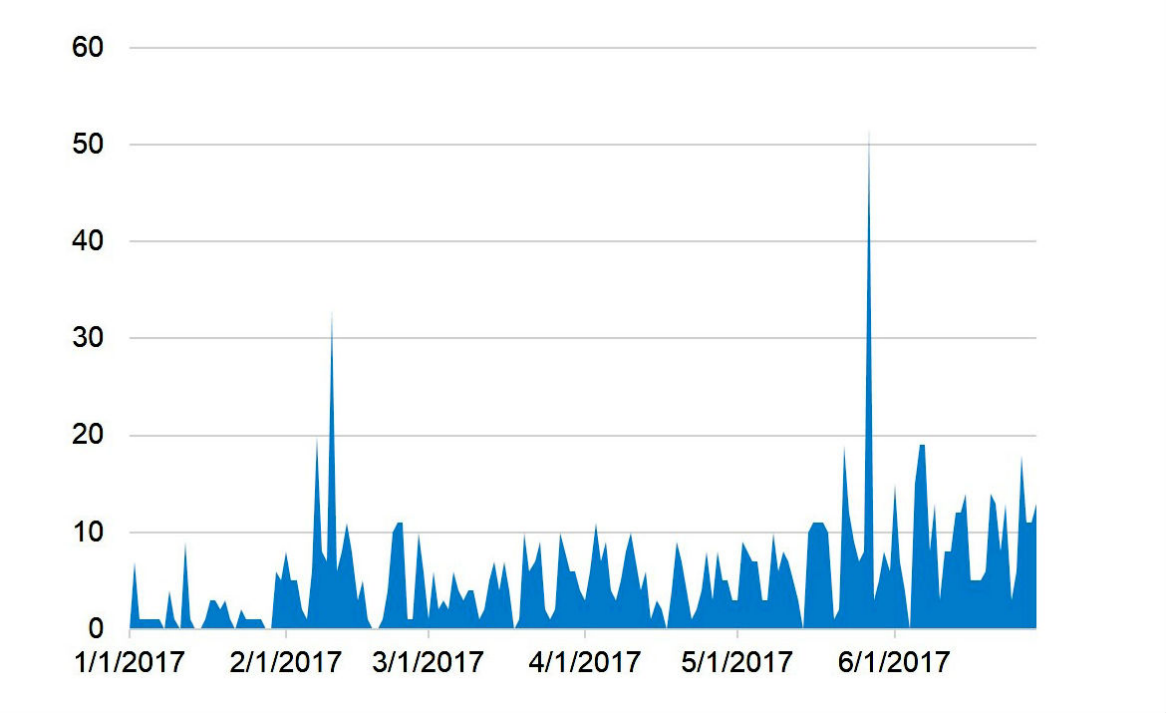
Number of visitors per day to the MyHEART website from January 1, 2017 to June 29, 2017.

Among new users, the bounce rate was 77%, while the bounce rate among returning users was 49%. Most users spent a short time on the site (0 to 10 seconds), but the time among the remaining users was approximately evenly distributed between 11 and 1800 seconds. The page with the largest number of views among new users was “What do the blood pressure numbers mean?” (eg, understanding blood pressure values). Interestingly, among new users who accessed that page, the majority (89.8%, 495/551 of page views) accessed the page by conducting an organic search; new users spent an average of 2 minutes and 32 seconds on this page. Across all viewers, the most viewed content was the “What do the blood pressure numbers mean?” (55.41%, 604/1090 views), followed by the MyHEART website home page (47.06%, 513/1090 views). The spike in website visits noted during February and May 2017 (Figure 2) likely reflects stages of the MyHEART website dissemination plan and viewers directly accessing the MyHEART website via academic center emails, newsletters, and media inquiries.

## Discussion

### Principal Findings

The MyHEART website and corresponding toolkit were successfully developed with diverse young adult, community, and academic stakeholders. The website can provide young adults with evidence-based hypertension information to support their self-management goals. The corresponding toolkit can support clinicians’ efforts to share knowledge about hypertension with young adults and offer counseling about behavior change. The authors successfully engaged clinical staff and their patients across healthcare systems and are actively working to engage young adults in the community (with limited healthcare access). The MyHEART website’s accessibility on mobile platforms helps target the young adult population. However, the authors are learning how to increase the duration of engagement of young adults on the website. For example, an interactive functionality is in development with the goal of increasing the length of time young adults use the website and acquire hypertension information.

### Limitations

We recognize that the website and toolkit were created in English, limiting access to young adults and clinicians who are not fluent in English. However, our team plans to make these materials available in Spanish. The website also currently lacks interactivity, but we plan to add this in the near future. Finally, we did not conduct a comparative analysis with other technology or programs. We are continuing to expand our dissemination activities and will develop multidimensional interventions in the future.

### Conclusions

In collaboration with young adults, health systems, and community stakeholders, the MyHEART young adult website is a portable resource to provide evidence-based information to a hard-to-reach population. The MyHEART website and toolkit provide resources for patients, clinicians, and healthcare organizations to improve hypertension control in young adults

## Appendix

Multimedia Appendix 1 Selected screenshots of the MyHEART website.

Multimedia Appendix 2 Sample of detailed notes from CARDS Lay Advisory Group meeting on MyHEART's architectural structure.

Multimedia Appendix 3 Examples of promotional materials for healthcare providers in the MyHEART toolkit.

## Abbreviations

AHA: American Heart Association
AHRQ: Agency for Healthcare Research and Quality
CARDS: Community Advisors on Research Design and Strategies
CDC: Centers for Disease Control
HIP: Health Innovation Program
IRB: Institutional Review Board
MyHEART: My Hypertension Education and Reaching Target
NIH: National Institutes of Health
WCHQ: Wisconsin Collaborative for Healthcare Quality
WINRS: Wisconsin Network for Research Support
