# Supplementary material for: My Hypertension Education and Reaching Target (MyHEART): Development and Dissemination of a Patient-Centered Website for Young Adults with Hypertension
Source: JMIR Cardio. 2017 Sep 25;1(2):e5. doi: 10.2196/cardio.8025 (PMC5898439; doi:10.2196/cardio.8025)
Supplement: Multimedia Appendix 1 [file cardio_v1i2e5_app1.pdf]

MyHEART home page (http://www.MyHeartMyChoice.org)

Home About My Heart My Choice My Support

WHAT IS HIGH BLOOD PRESSURE?

Learn about high blood pressure

READ MORE

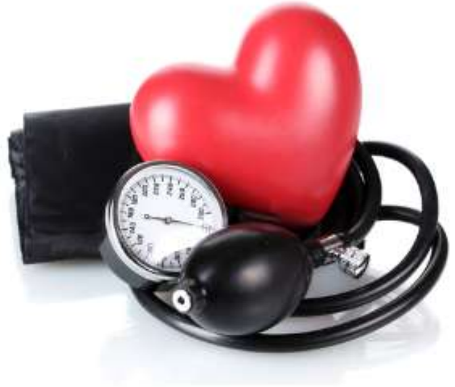

MY HEART

Learn About High Blood Pressure

- What is blood pressure?
- What do the numbers mean?
- Am I at risk for high blood pressure?
- How can I check my blood pressure?
- ...and other topics available

MY CHOICE

Healthy Choices You Can Make

- Manage Stress
- Eat Heart-Healthy Foods
- Limit Sodium (Salt)
- Quit Smoking
- ...and other topics available

MY SUPPORT

Up-to-Date Resources To Learn More

- Taking Your Blood Pressure
- Questions for Your Doctor
- Choosing a Healthy Diet
- Alcohol & Tobacco
- Additional Support

Welcome to MyHEART

Helping young adults learn more about high blood pressure

MyHEART stands for the My Hypertension Education and Reaching Target program. Our goal is to share information with young adults to help them live a healthier life, lower their blood pressure, and prevent heart disease. **MyHEART** was created with young adults and healthcare team members to address a broad range of topics. We welcome everyone to share this information with their family, friends, and other important people in their lives.

READ MORE

In the News

1. Prehypertension in pregnancy linked with issues for babies
   Pre-hypertension late in pregnancy may lead to babies who are underweight or stillborn.
2. Drinking soda can make you store more unhealthy fat
   Recent evidence shows that sugary drinks can lead to the accumulation of unhealthy fat over time.
3. Online weight management gets personal
   Use a free, research-based tool from the NIH to help you reach your weight loss and health goals.

VIEW ALL NEWS

My Heart section, Table of Contents page

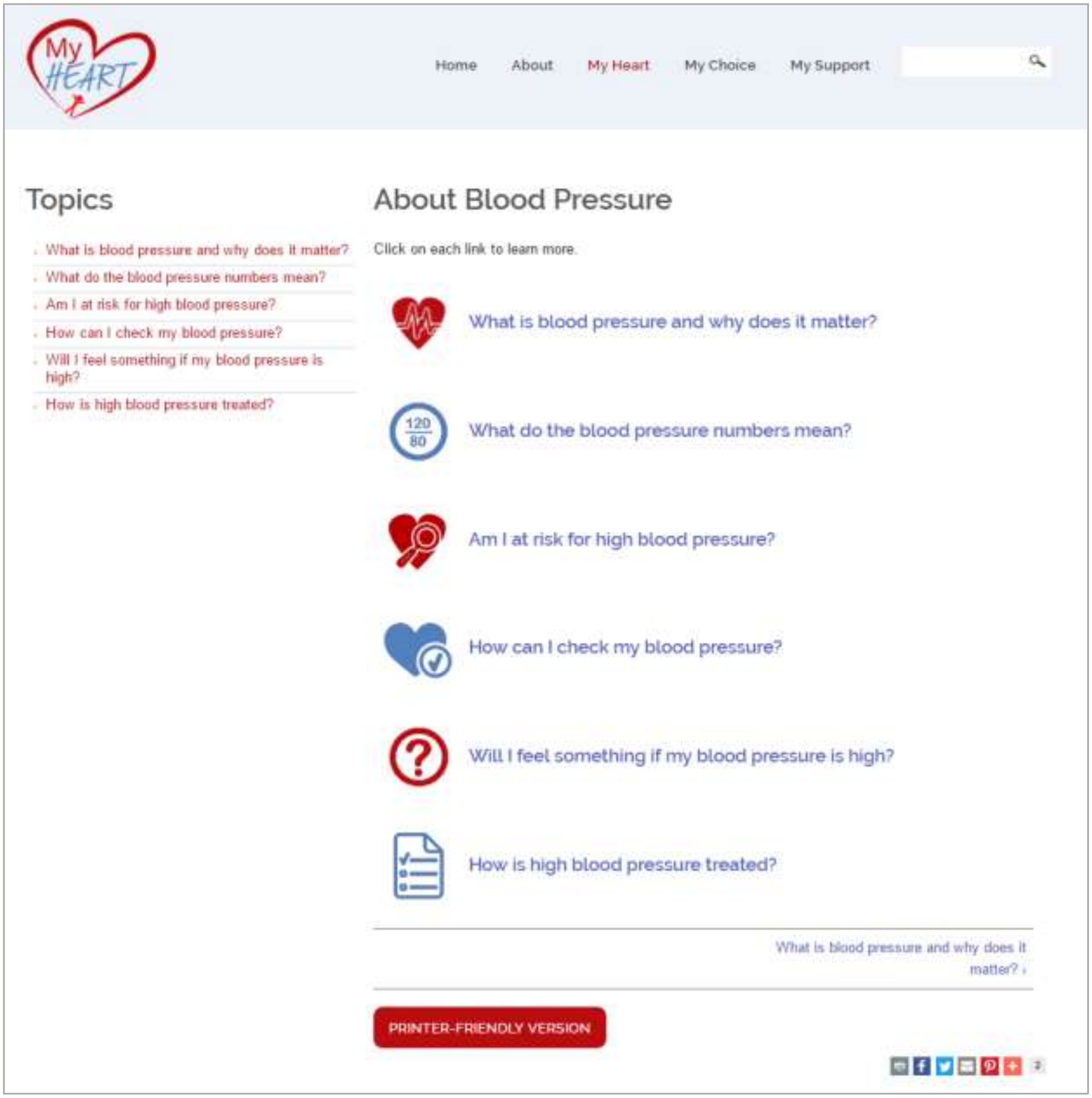

My Heart section, “What is blood pressure and why does it matter?” page

Topics

- What is blood pressure and why does it matter?
- What do the blood pressure numbers mean?
- Am I at risk for high blood pressure?
- How can I check my blood pressure?
- Will I feel something if my blood pressure is high?
- How is high blood pressure treated?

What is blood pressure and why does it matter?

Blood pressure is the force of blood pushing against the walls of your blood vessels.

This force is normally present with every heartbeat as the blood travels through your body. This force (pressure) is needed to carry blood to all the organs in your body, such as your kidneys, brain, stomach, arms, and legs.

High blood pressure, also called ***hypertension***, means that the pressure in your blood vessels stays higher than normal.

If your blood pressure stays high, it can become dangerous to your body.

Even as a young adult, high blood pressure can raise your risk of having serious health problems including a heart attack, stroke, kidney disease, or heart failure.

Taking steps to lower your blood pressure will help you live a healthier and longer life.

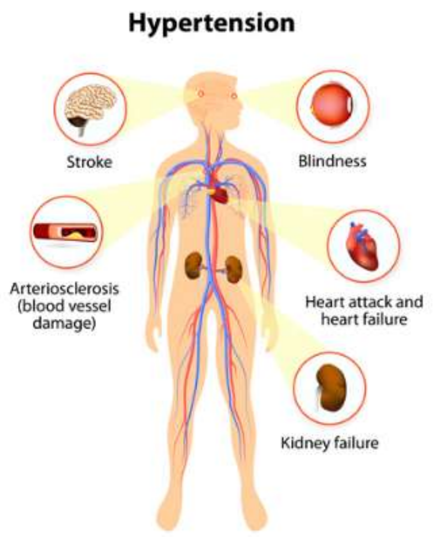

‹ About Blood Pressure

What do the blood pressure numbers mean? ›

PRINTER-FRIENDLY VERSION

My Choice section, Table of Contents page

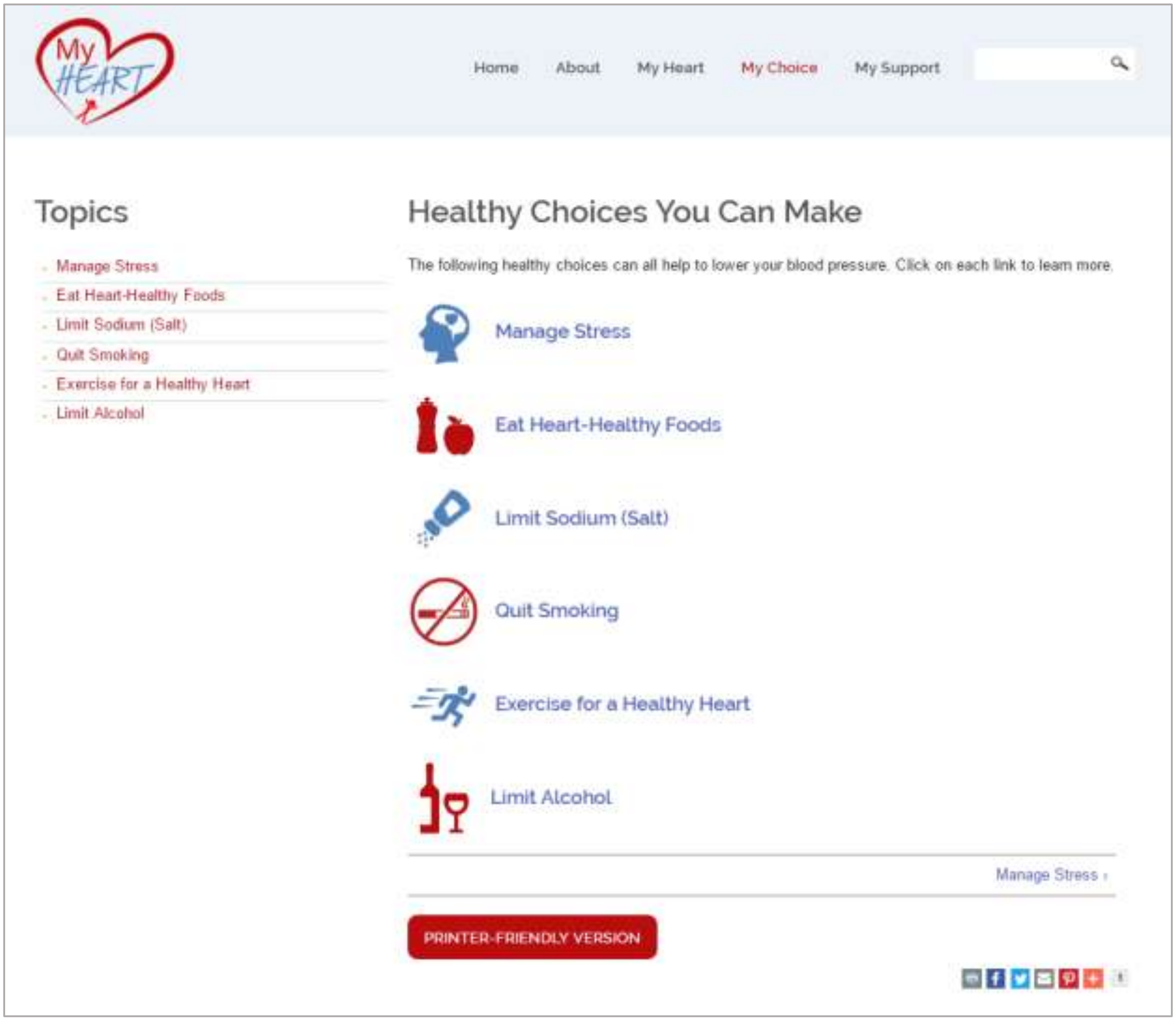

My Support section

Home About My Heart My Choice My Support

Up-to-Date Resources to Learn More

Taking Your Blood Pressure

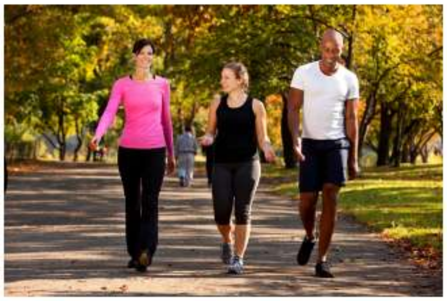

- How to Check Your Blood Pressure from the American Heart Association (This file will open in PDF format. You can download a free PDF reader here.)

- Printable Blood Pressure Log (This file will open in PDF format. You can download a free PDF reader here.)

Questions for Your Doctor

- Questions to ask your doctor about high blood pressure (This file will open in PDF format. You can download a free PDF reader here.)

- Questions for your doctor about blood pressure medications (This file will open in PDF format. You can download a free PDF reader here.)

Choosing a Healthy Diet

- Dietary Approaches to Stop Hypertension (DASH) eating plan

- MyPlate program

- Information on body weight

- Grocery list for healthy eating (This file will open in PDF format. You can download a free PDF reader here.)

Healthy Recipes

- Million Hearts® Heart-Healthy Recipes, Easy Meal Plans, and more

- MyFoodAdvisor® Recipes for Healthy Living

- American Heart Association Heart-Healthy Recipes

- NIH: National Heart, Lung, and Blood Institute's Delicious Heart Healthy Recipes

Exercise Plans to Get You Started

- American Heart Association's Getting Started - Tips for Long-term Exercise Success

- American Heart Association's Create Your Own Circuit Workout at Home

- American Heart Association's From the Couch to the Pavement - A Plan to Get You Moving
