# Supplementary material for: My Hypertension Education and Reaching Target (MyHEART): Development and Dissemination of a Patient-Centered Website for Young Adults with Hypertension
Source: JMIR Cardio. 2017 Sep 25;1(2):e5. doi: 10.2196/cardio.8025 (PMC5898439; doi:10.2196/cardio.8025)
Supplement: Multimedia Appendix 2 [file cardio_v1i2e5_app2.pdf]

CARDS® feedback on the overall page design of MyHEART's homepage; (Current/updated MyHEART home page: http://www.MyHeartMyChoice.org)

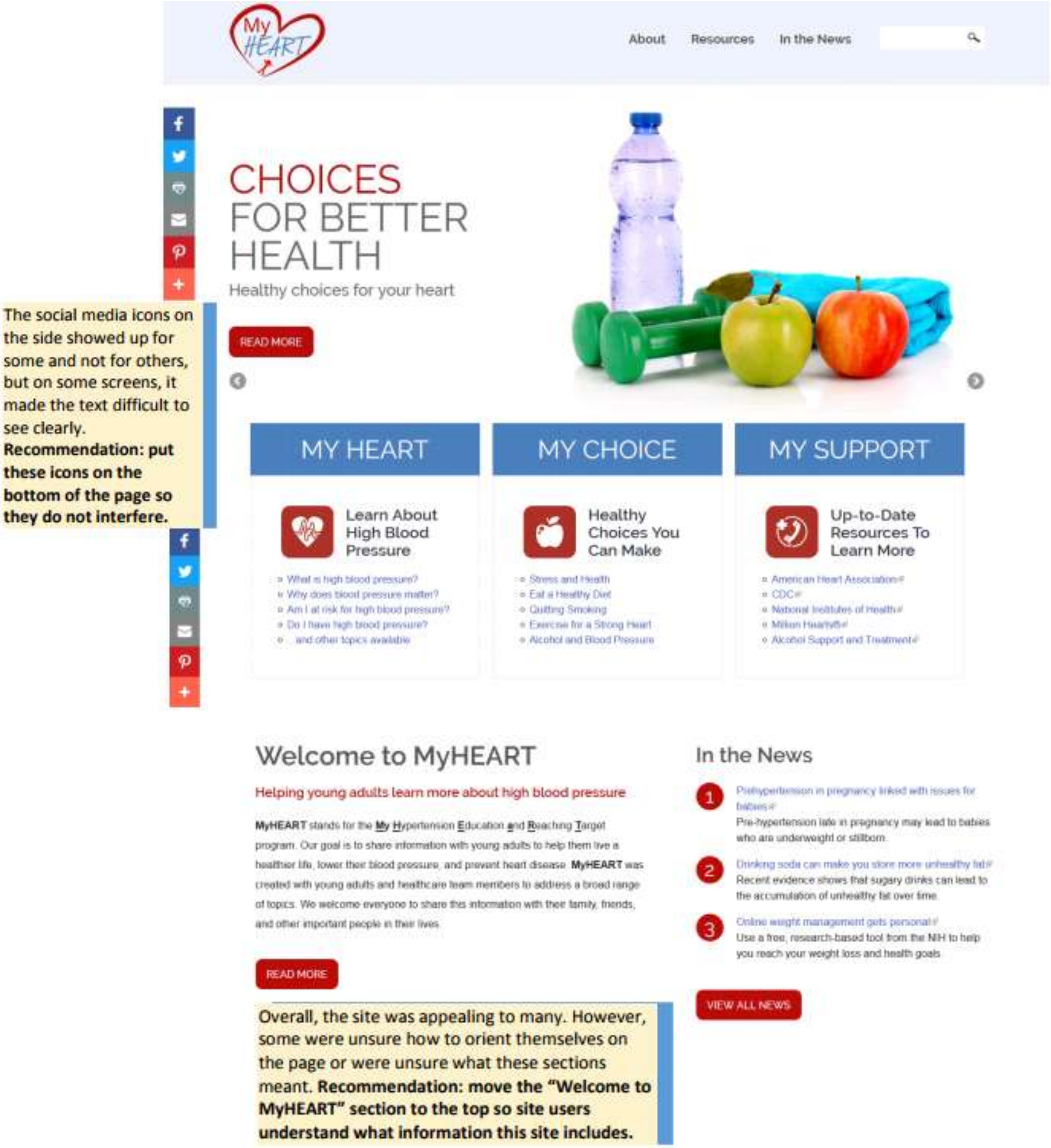

The social media icons on the side showed up for some and not for others, but on some screens, it made the text difficult to see clearly. **Recommendation: put these icons on the bottom of the page so they do not interfere.**

Overall, the site was appealing to many. However, some were unsure how to orient themselves on the page or were unsure what these sections meant. **Recommendation: move the "Welcome to MyHEART" section to the top so site users understand what information this site includes.**

CARDS® feedback on the design of an educational page; (Current/updated "How is high blood pressure treated?" page: http://myheartmychoice.org/HighBPTreatment)

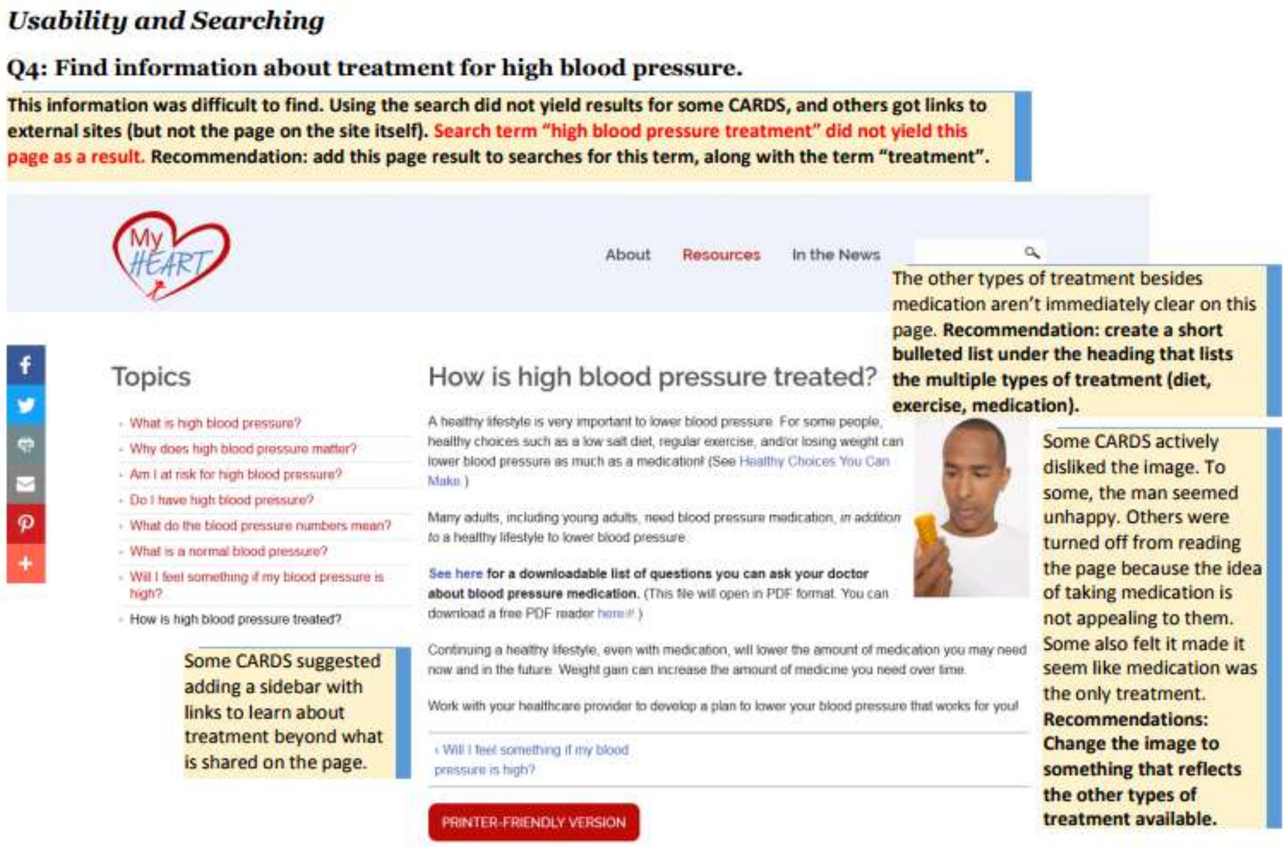

Usability and Searching

Q4: Find information about treatment for high blood pressure.

This information was difficult to find. Using the search did not yield results for some CARDS, and others got links to external sites (but not the page on the site itself). Search term "high blood pressure treatment" did not yield this page as a result. Recommendation: add this page result to searches for this term, along with the term "treatment".

About Resources In the News

Topics

- What is high blood pressure?
- Why does high blood pressure matter?
- Am I at risk for high blood pressure?
- Do I have high blood pressure?
- What do the blood pressure numbers mean?
- What is a normal blood pressure?
- Will I feel something if my blood pressure is high?
- How is high blood pressure treated?

How is high blood pressure treated?

A healthy lifestyle is very important to lower blood pressure. For some people, healthy choices such as a low salt diet, regular exercise, and/or losing weight can lower blood pressure as much as a medication! (See Healthy Choices You Can Make.)

Many adults, including young adults, need blood pressure medication, *in addition* to a healthy lifestyle to lower blood pressure.

See here for a downloadable list of questions you can ask your doctor about blood pressure medication. (This file will open in PDF format. You can download a free PDF reader here.)

Continuing a healthy lifestyle, even with medication, will lower the amount of medication you may need now and in the future. Weight gain can increase the amount of medicine you need over time.

Work with your healthcare provider to develop a plan to lower your blood pressure that works for you!

‹ Will I feel something if my blood pressure is high?

PRINTER-FRIENDLY VERSION

The other types of treatment besides medication aren't immediately clear on this page. **Recommendation: create a short bulleted list under the heading that lists the multiple types of treatment (diet, exercise, medication).**

Some CARDS actively disliked the image. To some, the man seemed unhappy. Others were turned off from reading the page because the idea of taking medication is not appealing to them. Some also felt it made it seem like medication was the only treatment. **Recommendations: Change the image to something that reflects the other types of treatment available.**

Some CARDS suggested adding a sidebar with links to learn about treatment beyond what is shared on the page.
