# Supplementary material for: My Hypertension Education and Reaching Target (MyHEART): Development and Dissemination of a Patient-Centered Website for Young Adults with Hypertension
Source: JMIR Cardio. 2017 Sep 25;1(2):e5. doi: 10.2196/cardio.8025 (PMC5898439; doi:10.2196/cardio.8025)
Supplement: Multimedia Appendix 3 [file cardio_v1i2e5_app3.pdf]

Printable MyHEART Poster in the MyHEART Toolkit (https://www.hipxchange.org/MyHEART)

1 IN 15 YOUNG ADULTS have high blood pressure

High blood pressure is dangerous for your body and can raise your risk of having serious health problems.

Learn how you can protect your heart

- FREE WEBSITE
- LEARN ABOUT HIGH BLOOD PRESSURE
- FIND HEALTHY CHOICES YOU CAN MAKE
- UP-TO-DATE RESOURCES YOU CAN TRUST

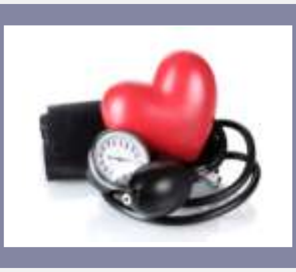

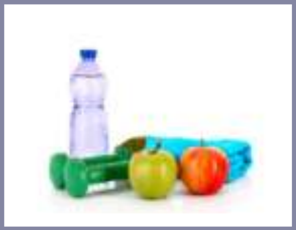

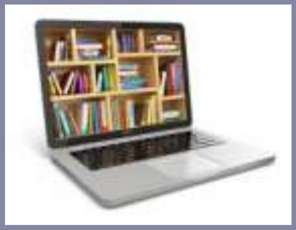

MyHeartMyChoice.org

@myheartmychoice.org

@MyHeartMyChoice

1 IN 15 YOUNG ADULTS have high blood pressure

High blood pressure is dangerous for your body and can raise your risk of having serious health problems.

Learn how you can protect your heart

- Free website
- Learn about high blood pressure
- Find healthy choices you can make
- Up-to-date resources you can trust

MyHeartMyChoice.org

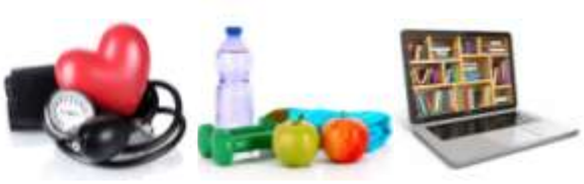

www.MyHeartMyChoice.org

Approximately 1 in 15 young adults have high blood pressure, increasing their risk of a future heart attack, stroke, congestive heart failure, and/or kidney disease.

Young adults have trouble controlling their blood pressure. They have the lowest rates of blood pressure control (achieving a blood pressure <140/90 mmHg) compared to other adult age groups.

Our goal is to share information with young adults to help them live a healthier life, lower their blood pressure, and prevent heart disease.

@MyHeartMyChoice

@MyHeartMyChoice.org
